# Access to Electronic Personal Health Records Among Patients With Multiple Chronic Conditions: A Secondary Data Analysis

**DOI:** 10.2196/jmir.7417

**Published:** 2017-06-02

**Authors:** Alexandra J Greenberg, Angela L Falisi, Lila J Finney Rutten, Wen-Ying Sylvia Chou, Vaishali Patel, Richard P Moser, Bradford W Hesse

**Affiliations:** 1 Mayo Clinic Rochester, MN United States; 2 Behavioral Research Program National Cancer Institute Bethesda, MD United States; 3 Office of the National Coordinator US Department of Health and Human Services Washington, DC United States

**Keywords:** electronic personal health information, electronic health records, patient engagement, multiple chronic conditions

## Abstract

**Background:**

In the United States, national incentives for offering access to electronic personal health records (ePHRs) through electronic means are geared toward creating a culture of patient engagement. One group of patients who stand to benefit from online access to ePHRs is the growing population with multiple chronic conditions (MCC). However, little is known about the current availability and use of ePHRs and patient portals among those managing MCC.

**Objective:**

The aim was to determine the associations between number of chronic conditions and sociodemographic characteristics and usage of ePHRs, and to assess how the public’s use of ePHRs varies across subpopulations, including those with MCC.

**Methods:**

This study used data collected from the 2014 Health Information National Trends Survey (HINTS), and assessed differences in use of ePHRs between those with and without MCC (N=3497) using multiple logistic regression techniques. Variables associated with health care systems (insurance status, having a regular provider) and patient-reported self-efficacy were included in the statistical models.

**Results:**

Those with MCC (n=1555) had significantly higher odds of accessing their records three or more times in the past year compared to those reporting no chronic conditions (n=1050; OR 2.46, 95% CI 1.37-4.45), but the overall percentage of those with MCC using ePHRs remained low (371 of 1529 item respondents, 25.63% weighted). No difference in odds of accessing their records was found between those reporting one chronic condition (n=892) and those reporting none (n=1050; OR 1.02, 95% CI 0.66-1.58). Significant differences in odds of accessing ePHRs were seen between income and age groups (*P*<.001 and *P*=.05, respectively), and by whether respondents had a regular provider (*P*=.03).

**Conclusions:**

We conclude that ePHRs provide a unique opportunity to enhance MCC patient self-management, but additional effort is needed to ensure that these patients are able to access their ePHRs. An increase in availability of patient access to their ePHRs may provide an opportunity to increase patient engagement and support self-management for all patients and especially those with MCC.

## Introduction

Over the next 40 years, the United States is expected to face a significant and rapid growth in its population aged 65 and older due in large part to the aging of the baby boomer generation [1]. With this aging population comes an increase not only in the number of individuals living with chronic disease, but also in the number of individuals living with multiple chronic conditions (MCC) [2]. Currently, 25% of Americans have two or more concurrent chronic conditions; these conditions include both physical conditions (eg, cardiovascular disease, diabetes, and cancer) and psychological conditions (eg, depression and anxiety) [3]. Individuals with two or more chronic conditions account for approximately 93% of Medicare spending; these costs are projected to increase in the coming years [4-7].

The US Department of Health and Human Services emphasizes the importance of patient-facing informatics tools for improving patient self-management and encouraging patient engagement, as evidenced by the creation of the Office of the National Coordinator for Health Information Technology in 2009. Widespread adoption of health information technology tools by the general population has been slow, but it is likely that the demands associated with MCC may encourage adoption in the near future [8]. Among older patients with diabetes, for example, the GroupHealth Cooperative found that the highest rate of use of secure messaging with providers was among those with multiple chronic conditions [9].

Health literacy is also key to using these tools because those with MCC need to have higher levels of health literacy when using electronic personal health records (ePHRs) [10,11]. Patients presenting with MCC have complex treatment regimens, some of which may be in conflict with one another [2]. Care is often fragmented and spread across several different providers and clinics, making coordination of care an additional concern for patients with MCC [2]. Furthermore, the complexity of care for these patients also results in more opportunities for medical errors, often due to missing data and note-taking differences in clinical records [12-15]. Therefore, there is a need for a technologically enabled health care system that can remove the burden from clinical personnel by enabling patients to be more engaged in their own care, simultaneously removing obstacles for timely patient-provider communication and preemptively decreasing the probability of medical error away from the clinic. At the same time, however, care must be taken to ensure that systems are not increasing the burden on the provider’s time [16]. From the perspective of the chronic care model, this implies that utilizing these new technologies and their accompanying workflow modifications creates a “prepared health system” [17-19].

At the same time, those diagnosed with MCC face challenges with the self-management of their ongoing care. This may be a result of impairment of physical or cognitive function, complexity of treatment regimens, and poor patient-provider communication [20,21]. In addition, fragmented care delivery poses myriad problems from the patient perspective with regard to managing personal health information (PHI) because it often falls on the patients to bring their records to each of their providers and to ensure that all their medications and therapeutic decisions are communicated accurately across points of care [22-24]. These tasks require a great deal of self-efficacy from patients, which has been linked to clinical outcomes [25-27]. For care of patients with several comorbidities to be successful and to result in improved quality of life, there is a need to have “prepared patients”—that is, patients who are equipped with tools and knowledge that empower them to communicate effectively with their providers, understand and manage their various treatment regimens, feel self-efficacious to take on these tasks, and consolidate their complex health care histories into one place [28,29].

To effectively support care of patients with MCC over time, there is a need to have both a “prepared health care system” and a population of “prepared patients.” Two complementary tools for addressing both the concepts of the “prepared system” and of the “prepared patient” are (1) electronic health records (EHRs) on the clinical side, and (2) connected access to that information through electronic personal health records (ePHRs) or patient portals on the patient side [19,30,31]. These ePHRs have the potential to serve as powerful tools for patient self-management, leading to the hypothesis that use of these tools will increase patient activation and self-efficacy and, in turn, improved clinical outcomes [32-34]. Studies have shown that “activated” and engaged patients tend to have lower health care costs and better care experiences than their less “activated” peers [35,36]. ePHRs hold particular promise for those with MCC because they have the potential to allow for care coordination between providers, allow for secure patient-provider communication, appointment management, information consolidation, and prescription refilling [22,37]. However, many individuals with MCCs have multiple providers and may consequently have multiple EHRs, potentially resulting in further confusion [2,25].

Although adoption of ePHRs sets the stage for patient engagement, there are no current data regarding the actual utilization of these tools from the public at large, especially for populations of patients struggling with MCC. To address this evidentiary gap, we analyzed data from the 2014 administration of the Health Information National Trends Survey (HINTS). The HINTS program was initiated in 2001 by the National Cancer Institute to provide surveillance on the public’s use of health information in a rapidly changing communication environment [38]. It has become the de facto source of data on many of the health communication and informatics objectives included in the Department of Health and Human Services’ Healthy People 2010 and 2020 initiatives [39], and has provided a complementary source of data for clinical audiences on the ways in which the public’s use of communication sources may influence practice [40]. In this study, we sought to answer the following questions:

1. What are the associations between number of chronic conditions, sociodemographic characteristics, and usage of ePHRs?
2. How does the public’s reported use ePHRs vary across different subpopulations, including populations who report MCC?
3. How likely is it for different groups of adults to have reported that they accessed their ePHRs and, more specifically, does the likelihood of accessing ePHRs increase as the number of chronic conditions increases?

## Methods

### Study Population and Data Collection

For this study, we analyzed data from the 2014 iteration of the HINTS (HINTS 4, Cycle 4) collected via a self-administered mailed survey between July and November 2014. The probability-based sampling frame used a two-stage design to achieve a nationally representative sample of US adults aged 18 years and older. Briefly, this design first used a stratified sample of residential addresses from the United States Postal Service, from which one adult from each sampled household was randomly selected. Efforts were made to oversample minority populations and those living in central Appalachia. The final number of respondents for this survey cycle was 3677 (response rate=34.4%). All items included in the HINTS administration underwent at least two rounds of cognitive testing through an external testing service (Westat, Rockville, MD, USA) for validation []. Additional information about data collection for HINTS 4, Cycle 4 can be found in the corresponding methodology report [41].

### Outcome Measure

The main outcome of interest was use of ePHRs. Respondents were asked: “How many times did you access your PHI online through a secure website or app in the last 12 months?”, with responses categorized as “none,” “1 to 2 times,” “3 to 5 times,” “6 to 9 times,” and “10 or more times.” A dichotomous variable for accessing ePHR was created by dividing respondents into “no/low” use and “medium/high” use. This categorization was based on the fact that no significant difference was found between those who reported never having accessed their record and those who reported accessing it 1 to 2 times (data not shown), and that the median response of those who had accessed their ePHRs at least once was the “3 to 5 times” category. Thus, no/low use consisted of individuals who responded “none” and “1 to 2 times,” and the medium/high use group consisted of individuals who responded “3 to 5 times,” “6 to 9 times,” and “10 or more times.”

### Independent Variables

Sociodemographic variables included in the analyses were age, sex, race/ethnicity, education, employment status, and income level. Health care variables included health insurance status and having a regular health care provider. Other independent variables from the survey included in our models were participant access to the Internet or email and self-reported ratings of general health on a five-point scale (ranging from “poor” to “excellent”). Self-efficacy was assessed using an item asking respondents to rate their self-reported ability to take care of their own health on a five-point scale (ranging from “not at all confident” to “very confident”). Response options for these variables were collapsed from five to three [42-45].

Additional items that dealt with use of technology were examined as well. These included items inquiring about whether respondents used mobile phones/tablets and whether they used health-related apps on these devices, whether they had emailed their doctor in the past 12 months, level of confidence that PHI was safe, feelings about control over privacy of ePHRs, whether they had ever withheld information from a provider due to concerns about privacy, and whether they were concerned about security of information when sent electronically between providers.

Chronic conditions were self-reported as part of the survey administration. Two questions were used to assess chronic conditions. The first asked: “Has a doctor or other health professional ever told you that you had any of the following conditions: (1) diabetes or high blood sugar; (2) high blood pressure or hypertension; (3) a heart condition such as heart attack, angina, or congestive heart failure; (4) chronic lung disease, asthma, emphysema, or chronic bronchitis; (5) arthritis or rheumatism; and (6) depression or anxiety disorder?” The second asked: “Have you ever been diagnosed as having cancer?” For each respondent, the number of chronic conditions were totaled; individuals were then categorized as having zero, one, or two or more chronic conditions for analyses. For this study, MCC was defined as having two or more of these conditions.

### Data Analyses

Analyses were conducted using SAS-callable SUDAAN 11.0.0 (RTI International, Research Triangle Park, NC, USA) and SAS 9.3 (SAS Institute Inc, Cary, NC, USA), which allowed analyses to account for the complex sampling procedure and to incorporate the jackknife replicate weights used for variance estimation. Descriptive statistics and bivariate analyses using chi-square tests of association were conducted.

Modeling included multivariable logistic regression using the dichotomous ePHR access variable as an outcome and adjusting for both theoretically important and statistically significant sociodemographic and health care characteristics. We regressed ePHR use onto levels of chronic condition, relevant demographics, health insurance / provider status, general health, and self-reported ability to take care of one’s health. Analyses were restricted to those with Internet access or who owned a mobile phone. The final sample weight was used in all analyses to obtain population-level point estimates and model parameters. For each analysis, listwise deletion of subjects was used.

## Results

Analyses were restricted to those who responded to the set of questions for self-reporting of chronic conditions. Of the 3497 individuals who responded to items about chronic conditions, 1050 (43.14% weighted) reported having no chronic conditions, 892 (24.85% weighted) reported having one chronic condition, and 1555 (32.01% weighted) reported having two or more chronic conditions.

### Associations Between Patient Factors and Number of Chronic Conditions

The first set of analyses explored bivariate relationships between a series of relevant patient characteristics and number of reported chronic conditions. Results from these analyses are presented in Table 1. As expected, there was a significant positive relationship between age and number of chronic conditions reported such that only 60 of 1555 (9.76% weighted) in the 18 to 34 years age range reported having two or more chronic conditions, whereas 274 of 1555 (71.49% weighted) in the 75 years and older range reported two or more conditions (Table 1). Sex, age, race/ethnicity, education, income, having a regular provider, and having health insurance were all significantly associated with number of chronic conditions. In general, having two or more chronic conditions was associated with being older, having health insurance, having a regular provider, being less confident in taking care of themselves, reporting fair to poor health, and being less inclined to use the Internet or to use a mobile phone/tablet (Table 1). No significant difference was found between those who responded to the chronic conditions items and those who did not, save for a difference in self-reported general health (*P*=.02); however, these results are not clinically or contextually significant because of small numbers in individual cells.

**Table 1:** Associations between patient characteristics, online characteristics, and attitudes with number of chronic conditions (N=3497).

Respondent characteristics		Number of chronic conditions, n (weighted %)^a^			χ^2^ (*df*)	*P* value
		0	1	≥2		
Overall		1050 (39.8)	892 (26.0)	1555 (34.1)		
**Sex**					5.5 (2)	.007
	Female	624 (36.2)	543 (27.3)	920 (36.5)		
	Male	420 (43.5)	344 (25.2)	605 (31.3)		
**Age (years)**					59.5 (8)	<.001
	18-34	283 (64.6)	117 (25.4)	60 (10.0)		
	35-49	322 (48.1)	200 (24.2)	188 (27.8)		
	50-64	272 (30.9)	299 (28.9)	556 (40.3)		
	65-74	72 (14.9)	149 (27.0)	349 (58.1)		
	≥75	28 (6.3)	74 (21.4)	274 (72.2)		
**Race/ethnicity**					4.1 (8)	.001
	Hispanic	185 (41.9)	132 (28.5)	194 (29.6)		
	Non-Hispanic White	555 (38.7)	496 (26.1)	844 (35.2)		
	Non-Hispanic Black	142 (39.9)	123 (24.0)	252 (36.1)		
	Non-Hispanic other	94 (57.4)	56 (22.5)	85 (20.0)		
	Missing	74 (28.9)	85 (26.9)	180 (44.2)		
**Education**					12.1 (6)	<.001
	Less than high school	59 (27.5)	65 (27.0)	163 (45.6)		
	High school graduate	171 (38.1)	140 (23.0)	323 (38.9)		
	Some college	257 (34.9)	282 (27.0)	511 (38.1)		
	College graduate	534 (49.6)	377 (26.7)	500 (23.7)		
**Income (US$)**					8.3 (8)	<.001
	<$20,000	163 (31.5)	182 (27.0)	442 (41.5)		
	$20,000 to <$35,000	127 (29.1)	116 (22.1)	265 (48.8)		
	$35,000 to <$50,000	145 (40.6)	139 (26.7)	220 (32.7)		
	$50,000 to <$75,000	180 (38.2)	153 (26.7)	252 (35.0)		
	≥$75,000	421 (48.7)	295 (26.5)	349 (24.9)		
**Health insurance**					9.3 (2)	<.001
	Yes	872 (38.3)	768 (25.8)	1397 (35.9)		
	No	168 (51.6)	110 (27.2)	130 (21.2)		
**Regular provider**					50.1 (2)	<.001
	Yes	548 (32.4)	612 (25.1)	1256 (42.5)		
	No	494 (54.3)	266 (27.7)	268 (18.0)		
**Self-reported ability to take care of own health**					9.7 (4)	<.001
	Completely confident/very confident	787 (42.9)	629 (27.2)	890 (29.8)		
	Somewhat confident	224 (34.8)	231 (24.8)	518 (40.4)		
	A little confident/not at all confident	36 (25.9)	29 (19.2)	137 (54.9)		
**Self-reported general health**					52.0 (4)	<.001
	Excellent/very good	675 (53.3)	443 (26.4)	427 (20.3)		
	Good	301 (31.5)	345 (28.8)	672 (39.7)		
	Fair/Poor	69 (17.9)	97 (17.5)	443 (64.7)		
**Regular Internet use**					35.9 (2)	<.001
	Yes	923 (42.9)	709 (26.1)	1077 (31.0)		
	No	123 (25.3)	173 (25.4)	455 (49.2)		
**Accessed EHRs at least once**					0.5 (2)	.61
	Yes	284 (28.0)	250 (26.7)	371 (25.5)		
	No	757 (72.0)	630 (73.3)	1158 (74.5)		
**Frequency of EHR access**					5.6 (8)	<.001
	Never	757 (72.0)	630 (73.3)	1158 (74.5)		
	1-2 times	158 (15.4)	124 (14.2)	153 (9.1)		
	3-5 times	74 (7.5)	78 (7.4)	101 (7.6)		
	6-9 times	24 (2.2)	30 (3.4)	57 (3.7)		
	≥10 times	28 (2.9)	18 (1.6)	60 (5.1)		
**Use a mobile phone or tablet**					36.7 (2)	<.001
	Yes	848 (44.9)	610 (26.0)	854 (29.1)		
	No	185 (25.7)	256 (26.2)	638 (48.1)		
**Use health-related mobile phone/tablet apps**					0.7 (2)	.49
	Yes	297 (46.0)	204 (24.4)	295 (29.6)		
	No	522 (44.2)	388 (27.9)	516 (27.9)		
**Exchanged emails with provider(s)**					0.3 (2)	.76
	Yes	246 (42.0)	206 (25.7)	331 (32.3)		
	No	791 (39.6)	662 (26.1)	1179 (34.3)		
**Confidence that PHI is safe**					2.0 (2)	.11
	Very confident	207 (38.9)	178 (23.7)	389 (37.5)		
	Somewhat confident	534 (38.3)	473 (27.4)	809 (34.3)		
	Not confident	295 (44.4)	221 (24.9)	324 (30.8)		
**Control privacy of records**					3.3 (4)	.02
	Very confident	255 (34.5)	246 (26.8)	487 (38.7)		
	Somewhat confident	479 (39.2)	420 (26.0)	733 (34.7)		
	Not confident	307 (47.6)	215 (25.2)	302 (27.1)		
**Ever withheld information due to privacy concern**					0.4 (2)	.66
	Yes	160 (43.0)	128 (24.4)	222 (32.6)		
	No	882 (39.4)	754 (26.3)	1306 (34.2)		
**Concerned about security of information when sent between providers**					1.2 (4)	.32
	Very concerned	226 (41.9)	191 (25.9)	338 (32.3)		
	Somewhat concerned	510 (40.4)	431 (24.4)	756 (35.3)		
	Not concerned	305 (37.9)	259 (28.9)	433 (33.2)		

^a^ Percentages are weighted.

### Assessing Likelihood of Utilizing Personal Health Information Online

In the second set of analyses, we formulated a multivariable binomial logistic regression model based on a combination of model selection techniques (Hosmer-Lemeshow and Akaike information criterion). Results are presented in Table 2.

The first notable finding was that, even when controlling for the influence of other variables in the model, there was a strong and unique contribution from number of chronic conditions; those reporting two or more chronic conditions had significantly higher odds of reporting medium/high use of ePHRs (OR 2.55, 95% CI 1.36-3.71; Table 2). Once we controlled for numbers of chronic conditions, the relationship between age and ePHR use also persisted with those in the lowest age bracket having the greatest odds of accessing ePHRs (OR 3.81, 95% CI 1.53-9.52; Table 2). The independent relationship between income and ePHR use also persisted, with those earning more than US $75,000 having the highest odds for accessing ePHRs (OR 3.74, 95% CI 1.74-8.07; Table 2). Of the health care system-related variables included in the model, only the regular provider variable showed a significant relationship to ePHR access (OR 1.72, 95% CI 1.07-2.77; Table 2).

**Table 2:** Weighted multivariate logistic regression model of predictors of using electronic personal health records among those reporting having Internet access or who own a mobile phone (n=2941).

Predictors of use of electronic personal health records		OR (95% CI)	Beta (SE)	Adj Wald *F* (*df*)	*P* value
**Number of chronic conditions**				4.51 (2)	.02
	0	Ref	Ref		
	1	0.98 (0.60-1.59)	-0.02 (0.24)		
	≥2	1.88 (1.09-3.24)	0.63 (0.27)		
**Sex**				0.13 (1)	.72
	Male	Ref	Ref		
	Female	1.06 (0.77-1.45)	0.16 (0.16)		
**Age (years)**				2.05 (4)	.10
	≥75	Ref	Ref		
	65-74	1.80 (0.69-4.66)	0.59 (0.48)		
	50-64	2.39 (1.01-5.67)	0.87 (0.43)		
	35-49	2.68 (1.13-6.36)	0.98 (0.43)		
	18-34	3.23 (1.24-8.41)	1.17 (0.47)		
**Race/ethnicity**				0.98 (4)	.43
	Non-Hispanic White	Ref	Ref		
	Hispanic	0.62 (0.31-1.26)	-0.47 (0.35)		
	Non-Hispanic Black	0.90 (0.57-1.42)	-0.11 (0.23)		
	Non-Hispanic other	1.34 (0.70-2.55)	0.29 (0.32)		
	Missing	0.47 (0.14-1.54)	-0.76 (0.59)		
**Education**				1.35 (3)	.27
	Less than high school	Ref	Ref		
	High school graduate	1.22 (0.25-5.88)	0.20 (0.78)		
	Some college	1.51 (0.35-6.52)	0.41 (0.73)		
	College graduate	1.85 (0.41-8.31)	0.61 (0.75)		
**Income (US$)**				3.04 (4)	.03
	<$20,000	Ref	Ref		
	$20,000 to <$35,000	1.90 (0.81-4.47)	0.42 (-0.21)		
	$35,000 to <$50,000	2.75 (1.25-6.08)	0.39 (0.22)		
	$50,000 to <$75,000	1.89 (0.85-4.23)	0.40 (-0.16)		
	≥$75,000	3.17 (1.50-6.71)	0.37 (0.41)		
**Health insurance**				1.71 (1)	.20
	No	Ref	Ref		
	Yes	1.48 (0.81-2.71)	0.30 (-0.21)		
**Regular provider**				7.43 (1)	.01
	No	Ref	Ref		
	Yes	1.84 (1.17-2.88)	0.61 (0.22)		
**Self-reported ability to take care of own health**				0.21 (2)	.81
	A little confident/not at all confident	Ref	Ref		
	Somewhat confident	0.97 (0.40-2.34)	-0.03 (0.44)		
	Completely confident/very confident	1.14 (0.54-2.39)	0.13 (0.37)		
**Self-reported general health**				1.71 (2)	.19
	Excellent/very good	Ref	Ref		
	Good	1.40 (0.94-2.09)	0.34 (0.20)		
	Fair/Poor	1.04 (0.52-2.10)	0.04 (0.35)		
**Confidence that PHI is safe**				5.24 (2)	.01
	Not confident	Ref	Ref		
	Somewhat confident	1.99 (1.25-3.17)	0.69 (0.23)		
	Very confident	2.00 (1.21-3.31)	0.69 (0.25)		

## Discussion

This study analyzed data from a nationally representative sample of noninstitutionalized US adults to gain a better understanding of who might be reporting use of ePHRs. Our analysis focused on usage patterns from those with MCC, a specific patient population that stands to benefit greatly from effective implementation and usage of ePHRs. When offered access to their ePHRs, patients with MCC had significantly higher odds of accessing their record more frequently than those without MCC; however, overall usage among those with MCC was lower than their healthy counterparts not reporting a chronic condition (25.6% and 29.1%, respectively).

Patients with MCC require complex care that demands a great deal from both the health care system and the patient. ePHRs and associated patient portals hold great promise for improving care coordination, patient-provider communication, shared decision making, appointment management, information consolidation, and management of medication for those with multiple comorbidities if incorporated into the patient’s self-management routines on a regular basis [22,46]. However, there are barriers to accessing and adopting patient portals. Several recent studies have documented barriers, namely a lack of information about the availability of the portals and/or motivation to use them [47,48]. Furthermore, older adults, including those with chronic conditions, had more difficulty using ePHRs and patient portals than their middle-aged counterparts [49]. All these studies on access barriers, however, involved deployment of the ePHR and portals specifically for the study. In the general population, the update rates may be higher, as was shown in one investigation of proactive engagement versus passive delivery of ePHR access in primary care clinics [50].

In order to increase adoption of ePHRs among patients with MCC, there is a need to understand the current state of use especially among these individuals. Thus, we first examined the sociodemographic and health care-related characteristics associated with MCC and then looked at whether an association exists between amount of ePHR use and number of chronic conditions reported in particular. First, older age was significantly associated with a greater number of chronic conditions. As would be expected, the accumulation of chronic conditions mounts over the life span with the majority of Americans aged 75 years and older reporting two or more conditions. This is the same group of Americans that is vulnerable to being left behind as health care becomes increasingly digitally based. In fact, our data showed that the majority of people reporting two or more chronic conditions also reported not using the Internet on a regular basis. Further work is needed, however, to determine what components of ePHRs and patient portals would be of greatest utility in their day-to-day lives.

We also examined which populations reported online access to ePHRs. Here we found strong relationships between ePHR use and education, income, having health insurance, and having a regular provider. Intriguingly, we also identified a significant relationship between ePHR usage and number of chronic conditions such that patients reporting two or more chronic conditions reported twice the frequency of ePHR use compared to those who reported one or no chronic conditions. To control for those influences, we regressed ePHR usage on a set of potential predictors as included in our previous analyses. This revealed an independent, significant relationship between ePHR usage and number of chronic conditions reported regardless of age, education, income, and other variables included in the logistic regression. Specifically, those reporting two or more chronic conditions had more than two-fold increased odds of accessing their ePHRs as compared to those reporting no chronic conditions. Although some studies examining associations between patient portal use included comorbidities as part of their models, none looked specifically at the burden of MCC in association with amount of use [51-55]. One previous study has shown that patients diagnosed with two or more chronic conditions activated ePHR accounts at higher rates than their healthy counterparts [51], but to our knowledge, ours is the first study to examine population data relating use of ePHRs to number of chronic conditions.

This finding is evocative of what some have referred to as “the diagnosis effect”: once diagnosed with a disease, patients tend to be more proactive in monitoring the implications of their condition or conditions [56]. For patients with Internet access, ePHRs could potentially be utilized as an extension of the clinical encounter, helping patients with some unmet needs and even the coordination of care across medical specialties. In this sense, patients with MCC may be at the vanguard of ePHR adoption and may provide an illustration of how policies increasing patient access can help create a cultural shift toward patient activation. Indeed, the few studies emerging on the efficacy of online support tools have generally shown strong clinical effects on patient engagement and outcomes [52-55]. One recent investigation examined patient activation and utilization of ePHRs via a telephone survey and found no difference in patient activation; however, these individuals were not necessarily managing MCC nor were they recently diagnosed with a new condition, both of which could affect usage [57].

Limitations of this study are largely related to the nature of cross-sectional surveys; namely, somewhat lower response rates than prospective patient studies, the fact that causation could not be inferred between variables (such as the nature of the relationship between use of online access and ratings of self-efficacy), and the fact that the data, including diagnoses of chronic conditions, were self-reported by respondents. Additionally, the survey administered did not include a comprehensive measure of patient self-efficacy, limiting our analyses to a single item for that construct. Another limitation lies in the nature of the fragmented health care system in the United States in that a patient with MCC may see many different specialists and may have multiple portals and ePHRs, and may answer “yes” to having accessed either ePHR without giving further indication as to which provider they are referring. Individuals with MCCs are more likely to have high levels of contact with their health care providers and, therefore, may have more opportunities to learn about ePHR tools and to be prompted to register for and use them. Additionally, the data on frequency of use were collected in categories rather than counts themselves. We were also unable to examine what patients were doing with their portals (eg, managing appointments, viewing laboratory results). Furthermore, this administration of HINTS did not include items addressing the quality of the ePHR systems and the degree of access that the ePHRs allow (whether patients were able to manage appointments, prescriptions, etc), which can vary between software companies and products. Survey items did not collect data on where care was received (eg, community clinics, private practice), which could have an impact on whether ePHRs were available to respondents; we attempted to control for this using sociodemographic factors. Future administrations of the HINTS instrument will collect those variables.

Despite these limitations, there are some significant strengths associated with national surveillance data, such as those reported through HINTS. These data are reported from the general population rather than being restricted to hospital-only respondent pools, the response rates and coverage results and ability to generalize are more robust than those of online panel surveys, and the sampling weights are carefully derived post stratification to generate nationally representative population estimates. Furthermore, the sampling paradigm for the administration of HINTS presented here oversampled minorities as well as rural residents of Appalachia. Previous investigations have focused on single clinics or health care systems, and many investigations have been qualitative in design. Here, we were able to present the first analyses quantifying differences in offers of access to ePHRs between those with MCCs and those without, which have not previously been examined. Due to the richness of the HINTS data, we were able to include a variety of sociodemographic and health care variables in our models that other investigations have not been able to address, including those reported from the patient’s perspective (ie, self-rated general health and self-efficacy).

Our analyses revealed a strong relationship between the presence of MCC and reports of electronic access to ePHRs as portrayed through a national probability sample of American adults. Our data show that those with two or more conditions have higher odds of accessing their ePHRs more frequently, but the numbers themselves remain low and the differences, although statistically significant, may not be of clinical significance. Our examination of variables related to both clinical and patient characteristics show that, although the system-related components may be in place, additional effort will be necessary to ensure that patients with MCC are equipped to use their ePHRs.

Targeted interventions and emphasis on patient engagement with these tools in MCC populations could greatly impact clinical outcomes. Assessments of those living with MCC have indicated that they are receptive to Web-based and app-based interventions, if the tools and interfaces were tailored to them and addressed any health and technology literacy divides [31,58]. These tools not only have the potential to support patients, but also to allow clinicians to monitor MCC patient behaviors and compliance with the various recommendations and prescribed treatments. Future planned studies involving the next HINTS administration will focus on what patients are using these portals to do; how patients are accessing these records; whether there is an association between MCCs, online access to their ePHRs, and primary care use; how use of ePHRs among those with MCC affect coordination of care; and whether outcomes are improved among those with MCC who engage with their ePHRs.

## Abbreviations

EHR: electronic health records
ePHR: electronic personal health record
HINTS: Health Information National Trends Survey
MCC: multiple chronic conditions
PHI: personal health information
