# Correction: Access to Electronic Personal Health Records Among Patients With Multiple Chronic Conditions: A Secondary Data Analysis

**DOI:** 10.2196/39719

**Published:** 2022-06-20

**Authors:** Alexandra J Greenberg, Angela L Falisi, Lila J Finney Rutten, Wen-Ying Sylvia Chou, Vaishali Patel, Richard P Moser, Bradford W Hesse

**Affiliations:** 1 Mayo Clinic Rochester, MN United States; 2 Behavioral Research Program National Cancer Institute Bethesda, MD United States; 3 Office of the National Coordinator US Department of Health and Human Services Washington, DC United States

In “Access to Electronic Personal Health Records Among Patients With Multiple Chronic Conditions: A Secondary Data Analysis” (J Med Internet Res 2017;19(6):e188), the authors made the following updates.

The authors were notified of data errors in one of the Health Information National Trends Survey (HINTS) cycle datasets (HINTS 4, Cycle 4); the errors were in the weights provided for use in the analysis of these data [1]. Following the HINTS error notice [1], the authors reran analyses reported in Table 1 and Table 2. The originally published versions of these tables are in Multimedia Appendix 1 and Multimedia Appendix 2.

In rerunning analyses for Table 1, only one difference was found that resulted in a change in conclusion. Namely, the chi-squared analysis of “Confidence that PHI is safe” versus “Number of Chronic Conditions” was not significant in the updated analysis (*P*=.11). In the original analysis, the results were significant, with “Very Confident” more likely with two or more chronic conditions, and “Not Confident” more likely with no chronic conditions. Additionally, for “Accessed EHRs at least once” and “Frequency of EHR Access,” the original table used column percentages instead of row percentages; this has been corrected in the updated table.

In section “Associations Between Patient Factors and Number of Chronic Conditions” in the *Results*, the fourth sentence in the first paragraph originally read as follows:

In general, having two or more chronic conditions was associated with being older, having health insurance, having a regular provider, being less confident in taking care of themselves, reporting fair to poor health, being less inclined to use the Internet or to use a mobile phone/tablet, and in feeling more confident that their PHI is safe and controllable (Table 1).

It has been corrected as follows:

In general, having two or more chronic conditions was associated with being older, having health insurance, having a regular provider, being less confident in taking care of themselves, reporting fair to poor health, and being less inclined to use the Internet or to use a mobile phone/tablet (Table 1).

The following text from the *Discussion* was no longer accurate and has been removed from the corrected version of the article:

Additionally, HINTS included items addressing concerns about safety and privacy of electronic health information, which revealed that those with MCC reported slightly higher frequencies of believing that they were “very confident” in having control of the privacy of their records and that their PHI was safe with their providers. This could provide one explanation for the increased use of ePHR among those with MCC.

In rerunning analyses for Table 2, only one difference was found that resulted in a change in conclusion. Namely, for “Confidence that PHI is safe,” a significant association was found for “Very Confident” (OR 2.00, 95% CI 1.21-3.31; *P*=.01) and “Somewhat Confident” (OR 1.99, 95% CI 1.25-3.17) as compared to the reference of “Not Confident.” In the original analysis, neither was statistically significant. This change does not affect any of the text within the body of the manuscript. But it is a notable new conclusion, indicating that those who are more confident about the safety of their data are significantly more likely to use ePHR than those who are not confident about such security.

The corrected versions of Table 1 and Table 2 are below:

**Table 1:** Associations between patient characteristics, online characteristics, and attitudes with number of chronic conditions (N=3497).

Respondent characteristics		Number of chronic conditions, n (weighted %)^a^			χ^2^ (*df*)	*P* value
		0	1	≥2		
Overall		1050 (39.8)	892 (26.0)	1555 (34.1)		
**Sex**					5.5 (2)	.007
	Female	624 (36.2)	543 (27.3)	920 (36.5)		
	Male	420 (43.5)	344 (25.2)	605 (31.3)		
**Age (years)**					59.5 (8)	<.001
	18-34	283 (64.6)	117 (25.4)	60 (10.0)		
	35-49	322 (48.1)	200 (24.2)	188 (27.8)		
	50-64	272 (30.9)	299 (28.9)	556 (40.3)		
	65-74	72 (14.9)	149 (27.0)	349 (58.1)		
	≥75	28 (6.3)	74 (21.4)	274 (72.2)		
**Race/ethnicity**					4.1 (8)	.001
	Hispanic	185 (41.9)	132 (28.5)	194 (29.6)		
	Non-Hispanic White	555 (38.7)	496 (26.1)	844 (35.2)		
	Non-Hispanic Black	142 (39.9)	123 (24.0)	252 (36.1)		
	Non-Hispanic other	94 (57.4)	56 (22.5)	85 (20.0)		
	Missing	74 (28.9)	85 (26.9)	180 (44.2)		
**Education**					12.1 (6)	<.001
	Less than high school	59 (27.5)	65 (27.0)	163 (45.6)		
	High school graduate	171 (38.1)	140 (23.0)	323 (38.9)		
	Some college	257 (34.9)	282 (27.0)	511 (38.1)		
	College graduate	534 (49.6)	377 (26.7)	500 (23.7)		
**Income (US$)**					8.3 (8)	<.001
	<$20,000	163 (31.5)	182 (27.0)	442 (41.5)		
	$20,000 to <$35,000	127 (29.1)	116 (22.1)	265 (48.8)		
	$35,000 to <$50,000	145 (40.6)	139 (26.7)	220 (32.7)		
	$50,000 to <$75,000	180 (38.2)	153 (26.7)	252 (35.0)		
	≥$75,000	421 (48.7)	295 (26.5)	349 (24.9)		
**Health insurance**					9.3 (2)	<.001
	Yes	872 (38.3)	768 (25.8)	1397 (35.9)		
	No	168 (51.6)	110 (27.2)	130 (21.2)		
**Regular provider**					50.1 (2)	<.001
	Yes	548 (32.4)	612 (25.1)	1256 (42.5)		
	No	494 (54.3)	266 (27.7)	268 (18.0)		
**Self-reported ability to take care of own health**					9.7 (4)	<.001
	Completely confident/very confident	787 (42.9)	629 (27.2)	890 (29.8)		
	Somewhat confident	224 (34.8)	231 (24.8)	518 (40.4)		
	A little confident/not at all confident	36 (25.9)	29 (19.2)	137 (54.9)		
**Self-reported general health**					52.0 (4)	<.001
	Excellent/very good	675 (53.3)	443 (26.4)	427 (20.3)		
	Good	301 (31.5)	345 (28.8)	672 (39.7)		
	Fair/Poor	69 (17.9)	97 (17.5)	443 (64.7)		
**Regular Internet use**					35.9 (2)	<.001
	Yes	923 (42.9)	709 (26.1)	1077 (31.0)		
	No	123 (25.3)	173 (25.4)	455 (49.2)		
**Accessed EHRs at least once**					0.5 (2)	.61
	Yes	284 (28.0)	250 (26.7)	371 (25.5)		
	No	757 (72.0)	630 (73.3)	1158 (74.5)		
**Frequency of EHR access**					5.6 (8)	<.001
	Never	757 (72.0)	630 (73.3)	1158 (74.5)		
	1-2 times	158 (15.4)	124 (14.2)	153 (9.1)		
	3-5 times	74 (7.5)	78 (7.4)	101 (7.6)		
	6-9 times	24 (2.2)	30 (3.4)	57 (3.7)		
	≥10 times	28 (2.9)	18 (1.6)	60 (5.1)		
**Use a mobile phone or tablet**					36.7 (2)	<.001
	Yes	848 (44.9)	610 (26.0)	854 (29.1)		
	No	185 (25.7)	256 (26.2)	638 (48.1)		
**Use health-related mobile phone/tablet apps**					0.7 (2)	.49
	Yes	297 (46.0)	204 (24.4)	295 (29.6)		
	No	522 (44.2)	388 (27.9)	516 (27.9)		
**Exchanged emails with provider(s)**					0.3 (2)	.76
	Yes	246 (42.0)	206 (25.7)	331 (32.3)		
	No	791 (39.6)	662 (26.1)	1179 (34.3)		
**Confidence that PHI is safe**					2.0 (2)	.11
	Very confident	207 (38.9)	178 (23.7)	389 (37.5)		
	Somewhat confident	534 (38.3)	473 (27.4)	809 (34.3)		
	Not confident	295 (44.4)	221 (24.9)	324 (30.8)		
**Control privacy of records**					3.3 (4)	.02
	Very confident	255 (34.5)	246 (26.8)	487 (38.7)		
	Somewhat confident	479 (39.2)	420 (26.0)	733 (34.7)		
	Not confident	307 (47.6)	215 (25.2)	302 (27.1)		
**Ever withheld information due to privacy concern**					0.4 (2)	.66
	Yes	160 (43.0)	128 (24.4)	222 (32.6)		
	No	882 (39.4)	754 (26.3)	1306 (34.2)		
**Concerned about security of information when sent between providers**					1.2 (4)	.32
	Very concerned	226 (41.9)	191 (25.9)	338 (32.3)		
	Somewhat concerned	510 (40.4)	431 (24.4)	756 (35.3)		
	Not concerned	305 (37.9)	259 (28.9)	433 (33.2)		

^a^ Percentages are weighted.

**Table 2:** Weighted multivariate logistic regression model of predictors of using electronic personal health records among those reporting having Internet access or who own a mobile phone (n=2941).

Predictors of use of electronic personal health records		OR (95% CI)	Beta (SE)	Adj Wald *F* (*df*)	*P* value
**Number of chronic conditions**				4.51 (2)	.02
	0	Ref	Ref		
	1	0.98 (0.60-1.59)	-0.02 (0.24)		
	≥2	1.88 (1.09-3.24)	0.63 (0.27)		
**Sex**				0.13 (1)	.72
	Male	Ref	Ref		
	Female	1.06 (0.77-1.45)	0.16 (0.16)		
**Age (years)**				2.05 (4)	.10
	≥75	Ref	Ref		
	65-74	1.80 (0.69-4.66)	0.59 (0.48)		
	50-64	2.39 (1.01-5.67)	0.87 (0.43)		
	35-49	2.68 (1.13-6.36)	0.98 (0.43)		
	18-34	3.23 (1.24-8.41)	1.17 (0.47)		
**Race/ethnicity**				0.98 (4)	.43
	Non-Hispanic White	Ref	Ref		
	Hispanic	0.62 (0.31-1.26)	-0.47 (0.35)		
	Non-Hispanic Black	0.90 (0.57-1.42)	-0.11 (0.23)		
	Non-Hispanic other	1.34 (0.70-2.55)	0.29 (0.32)		
	Missing	0.47 (0.14-1.54)	-0.76 (0.59)		
**Education**				1.35 (3)	.27
	Less than high school	Ref	Ref		
	High school graduate	1.22 (0.25-5.88)	0.20 (0.78)		
	Some college	1.51 (0.35-6.52)	0.41 (0.73)		
	College graduate	1.85 (0.41-8.31)	0.61 (0.75)		
**Income (US$)**				3.04 (4)	.03
	<$20,000	Ref	Ref		
	$20,000 to <$35,000	1.90 (0.81-4.47)	0.42 (-0.21)		
	$35,000 to <$50,000	2.75 (1.25-6.08)	0.39 (0.22)		
	$50,000 to <$75,000	1.89 (0.85-4.23)	0.40 (-0.16)		
	≥$75,000	3.17 (1.50-6.71)	0.37 (0.41)		
**Health insurance**				1.71 (1)	.20
	No	Ref	Ref		
	Yes	1.48 (0.81-2.71)	0.30 (-0.21)		
**Regular provider**				7.43 (1)	.01
	No	Ref	Ref		
	Yes	1.84 (1.17-2.88)	0.61 (0.22)		
**Self-reported ability to take care of own health**				0.21 (2)	.81
	A little confident/not at all confident	Ref	Ref		
	Somewhat confident	0.97 (0.40-2.34)	-0.03 (0.44)		
	Completely confident/very confident	1.14 (0.54-2.39)	0.13 (0.37)		
**Self-reported general health**				1.71 (2)	.19
	Excellent/very good	Ref	Ref		
	Good	1.40 (0.94-2.09)	0.34 (0.20)		
	Fair/Poor	1.04 (0.52-2.10)	0.04 (0.35)		
**Confidence that PHI is safe**				5.24 (2)	.01
	Not confident	Ref	Ref		
	Somewhat confident	1.99 (1.25-3.17)	0.69 (0.23)		
	Very confident	2.00 (1.21-3.31)	0.69 (0.25)		

In addition, the corresponding author's email address has been changed to worisek.alexandra@gmail.com, as the author is no longer affiliated with Mayo Clinic College of Medicine and Science.

The correction will appear in the online version of the paper on the JMIR Publications website on June 20, 2022, together with the publication of this correction notice. Because this was made after submission to PubMed, PubMed Central, and other full-text repositories, the corrected article has also been resubmitted to those repositories.

## Appendix

Multimedia Appendix 1 Originally published Table 1.

Multimedia Appendix 2 Originally published Table 2.

## Abbreviations

HINTS: Health Information National Trends Survey
