# Supplementary material for: Correction: Access to Electronic Personal Health Records Among Patients With Multiple Chronic Conditions: A Secondary Data Analysis
Source: J Med Internet Res. 2022 Jun 20;24(6):e39719. doi: 10.2196/39719 (PMC9254044; doi:10.2196/39719)
Supplement: Multimedia Appendix 1 [file jmir_v24i6e39719_app1.pdf]

Table 1. Associations between patient characteristics, online characteristics, and attitudes with number of chronic conditions (N=3497).

Respondent characteristics		Number of chronic conditions, n (weighted %)[a]			χ^2 (df)	*P*
		0	1	≥2		
Overall		1050 (43.1)	892 (24.9)	1555 (32.0)		
**Sex**					10.5 (2)	<.001
	Female	624 (38.5)	543 (25.9)	920 (35.6)		
	Male	420 (48.1)	344 (24.0)	605 (27.9)		
**Age (years)**					57.3 (8)	<.001
	18-34	283 (66.4)	117 (23.9)	60 (9.8)		
	35-49	322 (47.7)	200 (24.6)	188 (27.7)		
	50-64	272 (30.9)	299 (30.0)	556 (42.1)		
	65-74	72 (14.1)	149 (27.4)	349 (58.6)		
	≥75	28 (7.1)	74 (21.4)	274 (71.5)		
**Race/ethnicity**					5.5 (8)	<.001
	Hispanic	185 (46.8)	132 (27.4)	194 (25.9)		
	Non-Hispanic white	555 (41.5)	496 (25.1)	844 (33.4)		
	Non-Hispanic black	142 (47.7)	123 (21.5)	252 (30.8)		
	Non-Hispanic other	94 (62.5)	56 (21.3)	85 (16.2)		
	Missing	74 (28.0)	85 (25.7)	180 (46.4)		
**Education**					15.8 (6)	<.001
	Less than high school	59 (26.6)	65 (26.1)	163 (47.3)		
	High school graduate	171 (41.3)	140 (21.2)	323 (37.5)		
	Some college	257 (37.2)	282 (27.1)	511 (35.7)		
	College graduate	534 (53.7)	377 (24.6)	500 (21.7)		
**Income (US$)**					6.1 (8)	<.001
	<$20,000	164 (33.3)	181 (26.7)	443 (40.0)		
	$20,000 to <$35,000	126 (36.5)	115 (20.0)	264 (43.4)		
	$35,000 to <$50,000	144 (42.3)	136 (26.9)	222 (30.8)		
	$50,000 to <$75,000	181 (42.7)	156 (25.6)	253 (31.7)		
	≥$75,000	421 (21.3)	297 (24.6)	346 (24.1)		
**Health insurance**					4.7 (2)	.01
	Yes	872 (41.8)	768 (25.0)	1397 (33.2)		
	No	168 (54.0)	110 (23.3)	130 (22.7)		
**Regular provider**					43.3 (2)	<.001
	Yes	548 (35.6)	612 (24.0)	1256 (40.4)		

	No	494 (57.0)	266 (26.1)	268 (16.9)		
**Self-reported ability to take care of own health**					8.8 (4)	<.001
	Completely confident/very confident	787 (46.5)	629 (26.0)	890 (27.5)		
	Somewhat confident	224 (36.5)	231 (24.3)	518 (39.2)		
	A little confident/not at all confident	36 (30.7)	29 (14.1)	137 (55.2)		
**Self-reported general health**					61.0 (4)	<.001
	Excellent/very good	675 (56.5)	443 (24.8)	427 (18.7)		
	Good	301 (34.5)	345 (27.3)	672 (38.2)		
	Fair/Poor	69 (19.2)	97 (17.8)	443 (63.0)		
**Regular Internet use**					52.8 (2)	<.001
	Yes	923 (46.9)	709 (24.5)	1077 (28.6)		
	No	123 (24.2)	173 (26.7)	455 (49.1)		
**Accessed EHRs at least once**					0.9 (2)	.42
	Yes	284 (29.1)	250 (27.2)	371 (25.6)		
	No	757 (71.0)	630 (72.8)	1158 (74.4)		
**Frequency of EHR access**					7.9 (8)	*P* <.001
	Never	757 (71.0)	630 (72.3)	1158 (74.4)		
	1-2 times	158 (16.3)	124 (15.1)	153 (8.6)		
	3-5 times	74 (6.8)	78 (6.7)	101 (8.1)		
	6-9 times	24 (2.2)	30 (3.6)	57 (4.1)		
	≥10 times	28 (3.8)	18 (1.9)	60 (4.8)		
**Use a mobile phone or tablet**					47.3 (2)	<.001
	Yes	848 (48.7)	610 (24.8)	854 (26.4)		
	No	185 (25.8)	256 (25.1)	638 (49.2)		
**Use health-related mobile phone/tablet apps**					0.6 (2)	.54
	Yes	97 (49.6)	204 (23.2)	295 (27.2)		
	No	522 (48.2)	388 (26.6)	516 (25.2)		
**Exchanged emails with provider(s)**					1.0 (2)	.37
	Yes	246 (47.3)	206 (23.7)	331 (29.0)		
	No	791 (42.3)	662 (25.2)	1179 (32.5)		
**Confidence that PHI is safe**					2.7 (4)	.04
	Very confident	207 (43.8)	178 (21.0)	389 (35.2)		
	Somewhat confident	534 (41.4)	473 (26.2)	809 (32.4)		
	Not confident	295 (47.0)	221 (24.6)	324 (28.4)		
**Control privacy of records**					3.5 (4)	.01
	Very confident	255 (38.1)	246 (25.3)	487 (36.6)		
	Somewhat confident	479 (42.3)	420 (24.8)	733 (32.9)		
	Not confident	307 (50.9)	215 (24.2)	302 (24.9)		

**Ever withheld information due to privacy concern**					0.2 (2)	.82
	Yes	160 (45.6)	128 (24.1)	222 (30.3)		
	No	882 (42.9)	754 (24.9)	1306 (32.2)		
**Concerned about security of information when sent between providers**					1.1 (4)	.39
	Very concerned	226 (46.6)	191 (23.9)	338 (29.5)		
	Somewhat concerned	510 (43.4)	431 (23.5)	756 (33.1)		
	Not concerned	305 (40.7)	259 (27.3)	433 (31.9)		

[a]Percentages are weighted.
