# Supplementary material for: Correction: Access to Electronic Personal Health Records Among Patients With Multiple Chronic Conditions: A Secondary Data Analysis
Source: J Med Internet Res. 2022 Jun 20;24(6):e39719. doi: 10.2196/39719 (PMC9254044; doi:10.2196/39719)
Supplement: Multimedia Appendix 2 [file jmir_v24i6e39719_app2.pdf]

Table 2. Weighted multivariate logistic regression model of predictors of using electronic personal health records among those reporting having Internet access or who own a mobile phone (n=2941).

Predictors of use of electronic personal health records		OR (95% CI)	Beta (SE)	Adj Wald *F* (df1,df2)	*P*
**Number of chronic conditions**				7.59 (2,49)	<.001
	0	Ref	Ref		
	1	1.02 (0.66-1.58)	0.02 (0.22)		
	≥2	2.25 (1.36-3.71)	0.81 (0.25)		
**Sex**				0.68 (1,49)	.41
	Male	Ref	Ref		
	Female	1.15 (0.82-1.60)	0.14 (0.17)		
**Age (years)**				2.59 (4,49)	.05
	≥75	Ref	Ref		
	65-74	1.96 (0.79-4.87)	0.67 (0.45)		
	50-64	2.42 (1.07-5.47)	0.88 (0.41)		
	35-49	2.63 (1.15-6.00)	0.97 (0.41)		
	18-34	3.81 (1.53-9.52)	1.34 (0.46)		
**Race/ethnicity**				0.76 (4,49)	.56
	Non-Hispanic white	Ref	Ref		
	Hispanic	0.78 (0.36-1.69)	–0.25 (0.39)		
	Non-Hispanic black	1.13 (0.71-1.80)	0.12 (0.23)		
	Non-Hispanic other	1.27 (0.62-2.58)	0.24 (0.35)		
	Missing	0.40 (0.10-1.61)	–0.91 (0.69)		
**Education**				2.21 (3,49)	.10
	Less than high school	Ref	Ref (		
	High school graduate	1.58 (0.47-5.33)	0.46 (0.60)		
	Some college	2.11 (0.66-6.76)	0.75 (0.58)		
	College graduate	2.44 (0.75-7.91)	0.89 (0.59)		
**Income (US$)**				6.16 (4,49)	<.001
	<$20,000	Ref	Ref		
	$20,000 to <$35,000	1.88 (0.79-4.48)	0.63 (0.43)		
	$35,000 to <$50,000	2.47 (1.13-5.41)	0.91 (0.39)		
	$50,000 to <$75,000	1.74 (0.71-4.26)	0.55 (0.45)		
	≥$75,000	3.74 (1.74-8.07)	0.89 (0.59)		
**Health insurance**				3.06 (1,49)	.09

	No	Ref	Ref		
	Yes	1.69 (0.92-3.08)	0.52 (0.30)		
**Regular provider**				5.29 (1,49)	.03
	No	Ref	Ref (		
	Yes	1.72 (1.07-2.77)	0.54 (0.24)		
**Self-reported ability to take care of own health**				0.73 (2,49)	.49
	A little confident/not at all confident	Ref	Ref		
	Somewhat confident	1.17 (0.44-3.14)	0.16 (0.49)		
	Completely confident/very confident	1.45 (0.58-3.60)	0.37 (0.45)		
**Self-reported general health**				1.56 (2,49)	.22
	Excellent/very good	Ref	Ref		
	Good	1.39 (0.95-2.03)	0.33 (0.19)		
	Fair/Poor	1.11 (0.59-2.08)	0.10 (0.31)		
**Confidence that PHI is safe**				2.00 (2,49)	.15
	Not confident	Ref	Ref		
	Somewhat confident	1.65 (0.97-2.83)	0.50 (0.27)		
	Very confident	1.74 (0.93-3.26)	0.55 (0.31)		
